# Letter to the editor: Statistical methodology critique and alternative approaches in H5Nx avian influenza seroprevalence study among French cats

**DOI:** 10.2807/1560-7917.ES.2025.30.15.2500237

**Published:** 2025-04-17

**Authors:** Yoshiyasu Takefuji

**Affiliations:** 1 Faculty of Data Science, Musashino University, Tokyo, Japan

**Keywords:** Firth logistic regression, nonparametric statistics, epidemiological methodology, seroprevalence analysis, statistical assumptions

**To the editor:** Bessière et al. conducted a seroprevalence study examining cats as sentinels of mammal exposure to H5Nx avian influenza viruses in France from December 2023 to January 2025 [1]. While their analysis using Firth logistic regression suggested that owned, non-hunting cats had 94% lower odds of being H5-seropositive than stray cats (odds ratio = 0.06; 95% confidence interval: 0–0.53; p = 0.01), several methodological concerns arise. The study's findings about cats in at-risk départements showing increased H5 exposure risk (p = 0.09) and the disproportionate sampling between at-risk (n = 23) and non-risk départements (n = 7) raise questions about the statistical approach.

Most critically, the application of Firth logistic regression in this study appears problematic due to multiple violations of its fundamental assumptions [2-7]. The method requires binary outcome, independent observations, no perfect separation, linearity of logit, absence of multicollinearity and outliers, sufficient sample size, and correct model specification – several of which are violated here. Of particular concern are the imbalanced sampling distribution across departments (23 vs seven) and potential interdependence of observations within geographical clusters, which directly contradict the independence assumption.

Given these limitations, alternative analytical approaches such as Spearman's correlation, Kendall's tau for monotonic patterns [8], or mutual information analysis for complex, nonmonotonic interactions would be more appropriate [9]. These methods are nonlinear nonparametric, incorporating three critical components: data distribution, the statistical relationships between variables, and the validation of statistical significance via p values, making them more suitable for analysing this complex epidemiological dataset.

Ultimately, a deep understanding of these tools and their assumptions is essential for conducting robust and accurate data analyses.
